# Patient and parent reported outcome measures in cleft lip and palate patients before and after secondary alveolar bone grafting

**DOI:** 10.1097/MD.0000000000009541

**Published:** 2017-12-29

**Authors:** Chun-Shin Chang, Christopher Glenn Wallace, Yen-Chang Hsiao, Ting-Chen Lu, Sue-Huei Chen, Fuan-Chiang Chan, Philip Kuo-Ting Chen, Jyh-Ping Chen, Chee-Jen Chang, M. Samuel Noordhoff

**Affiliations:** a Department of Chemical and Materials Engineering, College of Engineering, Chang Gung University; b Craniofacial Research Center, Department of Medical Research, Department of Plastic & Reconstructive Surgery and Department of Craniofacial Orthodontics, Chang Gung Memorial Hospital, Taoyuan; c Department of Psychology, National Taiwan University, Taipei; d Graduate Institute of Clinical Medical Sciences, School of Medicine, Chang Gung University, Taoyuan, Taiwan.

**Keywords:** alveolar bone cleft, alveolar bone graft, cleft lip, cleft palate, parent reported outcome measures, patient reported outcome measures, secondary bone grafting

## Abstract

European and Northern American healthcare authorities increasingly encourage the use of Patient Reported Outcome Measures (PROMs) that complement clinical and laboratory assessments to help holistically evaluate reconstructive outcomes. This is the first study to evaluate PROMs in cleft lip/palate patients who have, or have not, undergone secondary alveolar bone grafting (SABG).

A PROMs study was conducted; 40 consecutive consenting cleft lip/palate children between 8 and 14 years old were included. Twenty patients did, and 20 patients did not, have SABG. PROMs scores from children and parents in the 2 groups were compared.

Forty patients completed the trial. No significant differences in total score from the Chang Gung Short Form-15 (CGSF-15) were found between children and their parents. Children with SABG reported no more oral-nasal regurgitation than children without SABG, but tended to report more nasal obstruction. There were no statistically significant differences in parent reported outcomes between the 2 groups.

Cleft lip/palate patients who underwent SABG reported significantly less nasal regurgitation and more nasal obstruction compared to those patients who did not undergo SABG.

## Abstract 2

Supplemental Digital Content is available in the text

## 1 Introduction

Patient Reported Outcome Measures (PROMs) allow assessment directly from the patient of their health status and health-related quality of life. It is increasingly recognized that traditional biomedical outcomes such as clinical and laboratory measures need to be complemented by measures that focus on the concerns of patients in order to evaluate interventions and identify more appropriate forms of health care.^[1]^ European and North American national healthcare reviews indicate that PROMs will be increasingly used in the evaluation of health care technologies and healthcare services, and thus contribute to regulatory decision-making. In cleft lip/palate surgery, the majority of published outcomes have been evaluated from the perspective of clinicians and/or independent observers; few PROMs that are specifically tailored to measure cleft lip/palate outcomes have been used.^[2–7]^ The aim of this study is to compare such measures with our newly developed standardized instrument: “Chang Gung Short Form-15” (CGSF-15). This was designed to measure outcomes reported both by Patients (all children) with/without secondary alveolar bone grafting (SABG) and their Parents/Caregivers, so-called “Patient/Parent Reported Outcome Measures”. This is because both parents’ and patients’ concerns are important when it comes to evaluating children.^[7]^

## 2 Methods

**Figure 1:**
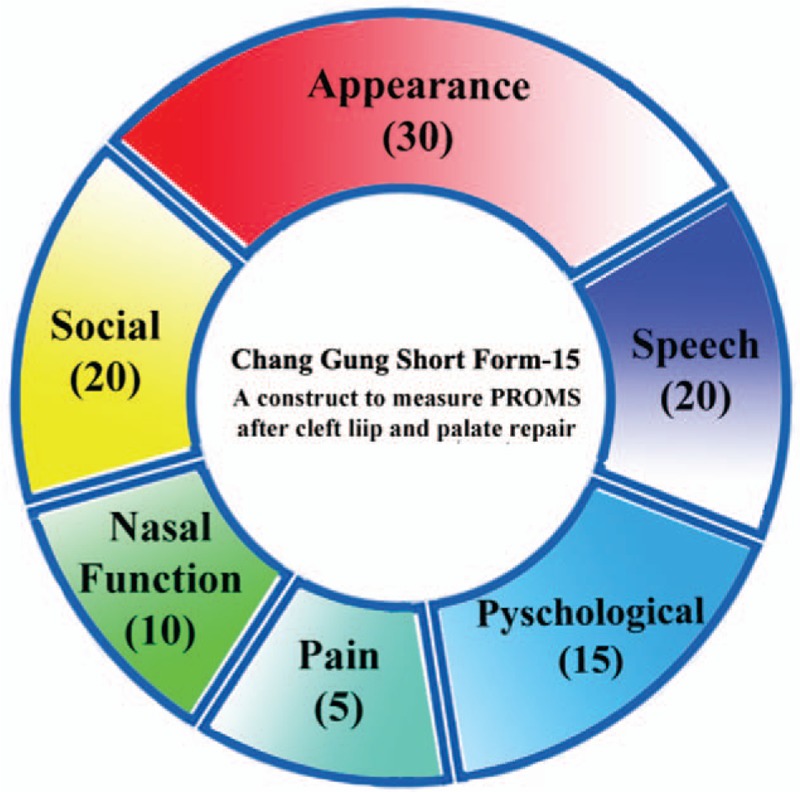
Domains of the Chang Gung Short Form-15.

This was a two-phase prospective study involving patients with complete unilateral cleft lip/palate repair and their parents. The first phase involved designing a weighted and validated Patient and Parent Reported Outcome Measures (PPROMs) instrument. After 3 pilot studies, a standardized, validated construct consisting of 5 weighted domains to evaluate PPROMs in this population was successfully established: the “Chang Gung Short Form-15”. The domains are: Appearance (30%), Speech (20%), Social (20%), Psychological (15%), Nasal Function (10%), and Pain (5%) (Fig. 1). The maximum score is 50 points, and points are weighted within each domain based on feedback from initial pilot studies. From these 5 domains, a comprehensive assessment of a patient's quality of life could be achieved; this formed the basis for the second phase of the study. The CGSF-15 is completed by patients and their parents, unobserved and unaided by the medical team. The pilot studies demonstrated in this population that an appropriate age for children to be able to complete the CGSF-15 competently is 8 years old and above (Supplemental information 1 and 2).

The CGSF-15 PPROMs instrument was developed after a comprehensive review of the literature and involvement of many patients, parents, and patient groups. Several draft questionnaires were developed and optimized, ultimately culminating in a final working questionnaire with 15 questions covering 5 domains. This new PPROM underwent 3 pilot tests during which monthly departmental conferences and discussions with patients/parents occurred to optimize the instrument continuously. Each item and its psychometric value were formulated based on interviews with patients and their parents/caregivers. After 1 year of continuous optimizations, the finalized PPROMs instrument was used to evaluate patients with unilateral complete cleft lip/palate who had or had not received SABG, and their parents.

### 2.1 Ethics

This trial was approved by the institutional review board (IRB) of Chang Gung Memorial Hospital (IRB 100-3763b). All methods were performed in accordance with the relevant guidelines and regulation. The date at which the ethics committee approved the study was July 1, 2012, the date that patient recruitment started was July 3, 2012 and the date that follow-up completed for the final patient was January 26, 2013. Twenty consecutive patients who had, and 20 consecutive patients who had not, received SABG were recruited during outpatient clinic visits at Chang Gung Memorial Hospital.

Inclusion criteria consisted of consenting unilateral complete cleft lip/palate patients and their parents; patients before and after SABG; and (patients aged between 8 and 14 years old. Exclusion criteria consisted of the presence of other craniofacial anomalies; patients without alveolar bone clefting.

### 2.2 Sample size calculation

STATA v9 (StataCorp LP, Texas) was used to determine recruitment needs to achieve adequate statistical power. The mean score of our pilot studies was 32.8 ± 12.4. Quinn et al^[8]^ reported that the minimum clinical importance on the 0 to 100 visual analog score for cosmesis was 15; this is reflected in the CGSF-15. Using the same SD with a power of 0.90 and Alpha of 0.05, the number of patients required was calculated to be 15 per group. We assumed that some questionnaires might be returned as invalid (e.g., provision of multiple answers, or no answer, on a single item) and therefore recruited 20 patients for both groups.

### 2.3 Statistical analyses

Statistical analyses were conducted with SPSS software (version 17.0; IBM Corporation, NY). CGSF-15 scores were collected and the 2 groups (patients with and without SABG) were compared. Differences in ordinal data were analyzed using the independent student *t*-test. Differences in nominal data were analyzed using Fisher's exact test. Statistical significance was defined if *P* was <.05. Data are presented as mean ± standard deviation unless otherwise stated.

## 3 Results

Forty patients and their parents completed the study. The age and sex of patients is tabulated (Table 1). All except 3 patients were treated entirely in our Craniofacial Center since birth. Of these 3, 1 underwent SABG elsewhere and 2 had been treated elsewhere since birth and had not undergone SABG.

**Table 1:**
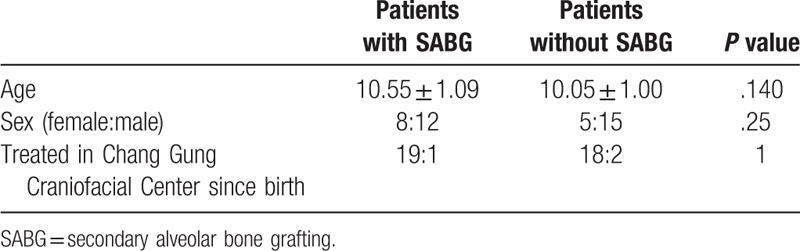
Patients’ sex and age.

According to questionnaires obtained from patients, CGSF-15 total scores from patients with and without SABG were not significantly different (patients with SABG = 33.8 ± 4.29 vs patients without SABG = 34.5 ± 4.43; *P* = .59). Furthermore, no significant differences were found in the scores between groups for the CGSF-15 domains (Appearance, Speech, Social, Psychological and Pain) (Table 2).

**Table 2:**
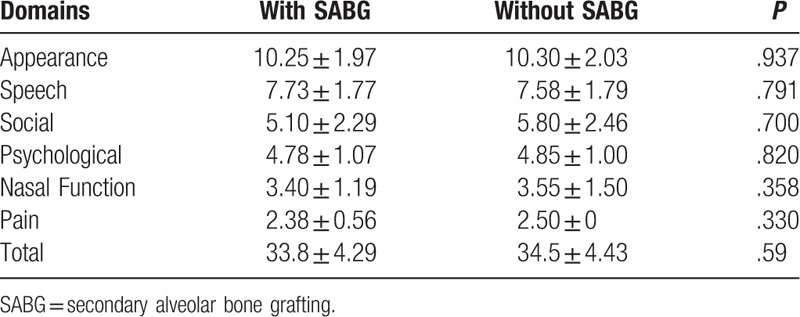
Summary of Chang Gung Short Form-15; questionnaires completed by the patient.

According to questionnaires obtained from parents/caregivers, patients with SABG and without SABG were not significantly different (with SABG 35.73 ± 4.30 vs without SABG 35.85 ± 4.69; *P* = .93). Furthermore, no significant differences were found in the scores between groups for the CGSF-15 domains (Appearance, Speech, Social, Psychological and Pain) (Table 3).

**Table 3:**
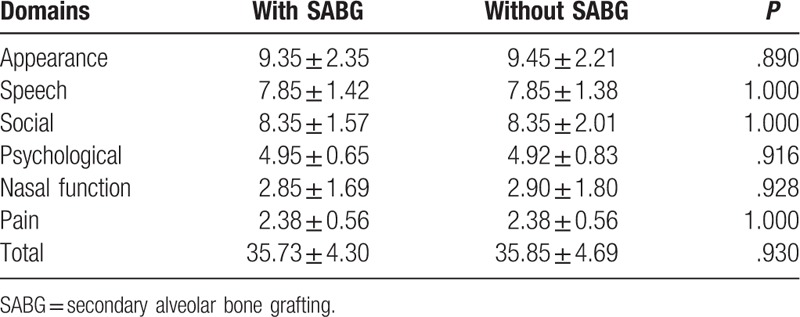
Summary of Chang Gung Short Form-15; questionnaires completed by the parents.

Analysis of individual questions revealed significant differences between the 2 groups only for patient reported nasal obstruction and nasal food regurgitation (Table 4). None of the patients with SABG reported nasal regurgitation, whereas 25% of the patients without SABG reported nasal regurgitation. Around 65% of the patients who had undergone SABG reported nasal obstruction, whereas 25% of the patients without SABG reported nasal obstruction.

**Table 4:**
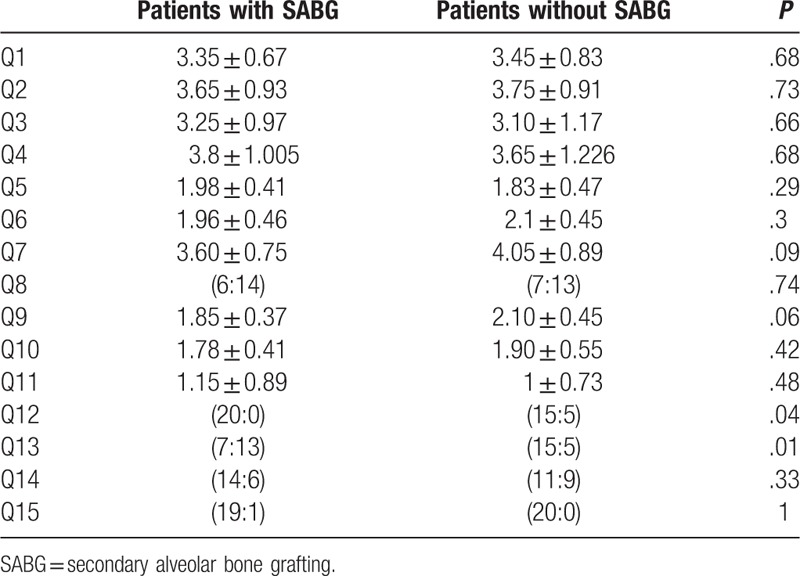
Questionnaires for children.

Analysis of the 15 questions for the parents revealed no statistically significant differences between groups (Table 5).

**Table 5:**
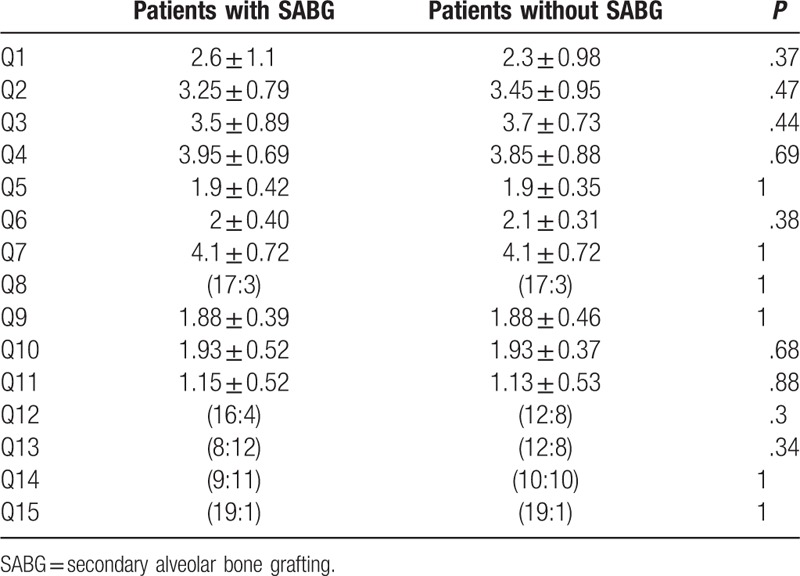
Questionnaires for parents.

## 4 Discussion

Patients with unilateral complete cleft lip/palate undergo several surgical procedures during their lifetimes, including cheiloplasty with or without primary rhinoplasty, with or without primary gingivoperiosteoplasty, palatoplasty, operations for velopharyngeal insufficiency, SABG, orthognathic surgery, and others. Each has diverse clinical goals and complex effects on patients’ lives. PROMs have previously been used in the field of cleft lip/palate^[3,5,9–11]^ but, to the best of our knowledge, none have addressed the effect of SABG on patients with unilateral complete cleft lip/palate, and on their parents.

Since Dr Samuel Noordhoof founded our Center more than 3 decades ago, we have evaluated cleft lip/palate patients’ outcomes based on clinical lip morphology,^[12]^ nasal morphology,^[13–15]^ lip scar,^[16]^ speech,^[17]^ complication rates,^[18]^ and facial growth.^[19]^ All were clinical studies based on data from the perspective of clinicians. However, a patient's (and their parents’) own perception of their surgical or orthodontic treatment outcome(s) and impact on their quality of life is of great importance for research by a craniofacial team to be holistic and integrated. This can only be achieved by means of PPROMs.

The U.S. Food and Drug Administration considers that patient-centered data can only be provided by PROMs, reflected by the increasing importance of PROMs in the plastic and reconstructive surgery literature.^[20]^ European and North American healthcare reviews indicate that PROMs will be increasingly used in the evaluation of health care technologies and healthcare services, and contribute to regulatory decision-making. Moreover, PROMs can provide data for better communication between healthcare providers (such as plastic surgeons and orthodontics) and patients. This will allow craniofacial centers to be more effective at addressing each problem that patients with cleft lip/palate may face.

An alveolar bone cleft is present in the majority of patients with cleft lip/palate. This bone defect destabilizes the maxillary arch and predisposes it to medial collapse. Successful SAGB can stabilize the maxillary arch, restore normal occlusion, provide a matrix for continued eruption of permanent teeth in this region, close a peri-alveolar oronasal fistula and allow improved periodontal health of the teeth adjacent to the cleft.^[21,22]^ Two-dimensional dental radiographs and three-dimensional computed tomography have been used to assess the success of SABG by physicians;^[21,23–33]^ however, whilst valuable, these do not measure the impact of SABG on health related quality of life for the patients. Currently the most frequently used questionnaires for cleft lip/palate patients are the Strengths and Difficulties Questionnaire, Childhood Experience Questionnaire and Satisfaction with Appearance Survey. These are useful for evaluating factors such as self-esteem, behavior, social support, and facial appearance,^[34]^ but do not address the aforementioned potential advantages of successful SABG. This study developed a newly devised CGSF-15 construct to evaluate PPROMs quantitatively in cleft lip/palate patients with and without SABG, and their parents. A further domain that we initially included during preliminary studies was patient reported dental satisfaction, but we found that this age group of patients did not consider this an important factor. We plan, however, to include this domain in future developments of the CGSF-15 aimed at older patients.

**Figure 2:**
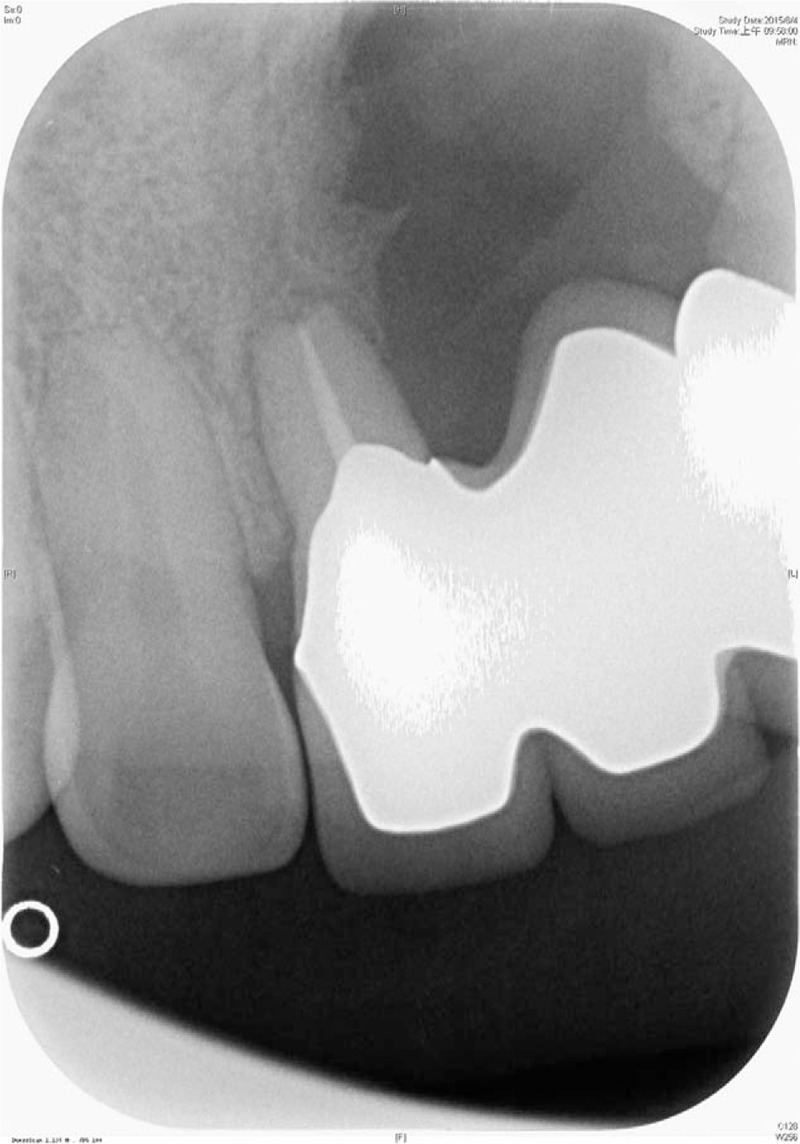
A 31 year-old male patient with unilateral complete cleft lip/palate. He did not have an alveolar bone graft. Radiographs showed peri-dental bone resorption and impending loss of the central incisor. The impact of clinical findings such as these are difficult to assess with questionnaires.

Limitations of PROMs in patients with SABG were found. It is difficult to address the following goals of SABG with questions: stabilization of the maxillary arch, provision of a matrix for continued eruption of permanent teeth in this region, closure of a peri-alveolar oronasal fistula and state of periodontal health of the teeth adjacent to the cleft. Patients without SABG but with residual cleft might experience peri-dental bone resorption and loosening of the teeth (Fig. 2).

In the present study, according to the questionnaires, none of the patients after SABG have residual oral-nasal fistula. However, they experienced more nasal obstruction compared to patients without SABG. The questionnaires for parents did not reveal this condition. This might be due to parents being less aware of the problems of oral-nasal fistula or nasal obstruction, if their children experienced them.

## 5 Conclusion

We believe that PROMs, and PPROMs for children, will play an increasingly significant role in decision-making regarding the future direction of health care delivery. PROMs/PPROMs provide opportunities to improve healthcare outcomes by giving decision makers data on how healthcare affects what patients are able to do, and how patients feel. Herein, we report the first PPROMs instrument that has allowed us to compare the outcomes of patients with unilateral complete CLP who have, or have not, undergone SABG from the perspective of both the children and their parents. Patients with SABGs did not report any nasal regurgitation but did report more frequent nasal obstruction compared with those who did not undergo SABG.

## Acknowledgment

We are grateful that this project was initiated by Fuan-Chiang Chan and helped by Prof. Sue-Huei Chen, Chee-Jen Chang, and Prof. Jyh-Ping Chen. We are grateful that this project was partly supported by a Chang Gung Memorial Hospital Research Grant (CMRPG5G0011).

## Supplementary Material

Supplemental Digital Content
