# Supplementary material for: Patient and parent reported outcome measures in cleft lip and palate patients before and after secondary alveolar bone grafting
Source: Medicine (Baltimore). 2017 Dec 29;96(52):e9541. doi: 10.1097/MD.0000000000009541 (PMC6393063; doi:10.1097/MD.0000000000009541)
Supplement: Supplemental Digital Content [file medi-96-e9541-s001.pdf]

Supplementary information: Questionnaire for children:

Q1. Does my upper lip symmetrical?

☐ Very Satisfied; ☐ Satisfied; ☐ Acceptable; ☐Unsatisfied; ☐Very unsatisfied

Q2. Does my upper lip is smooth and there is no uneven?

☐ Very Satisfied; ☐ Satisfied; ☐ Acceptable; ☐Unsatisfied; ☐Very unsatisfied

Q3. How is my upper lip scar?

☐ Very Satisfied; ☐ Satisfied; ☐ Acceptable; ☐Unsatisfied; ☐Very unsatisfied

Q4. Does my speech comprehensible?

☐ Very Satisfied; ☐ Satisfied; ☐ Acceptable; ☐Unsatisfied; ☐Very unsatisfied

Q5. How is my speech sound production?

☐ Very Satisfied; ☐ Satisfied; ☐ Acceptable; ☐Unsatisfied; ☐Very unsatisfied

Q6. Does my speech fluent?

☐ Very Satisfied; ☐ Satisfied; ☐ Acceptable; ☐Unsatisfied; ☐Very unsatisfied

Q7. How is my social interaction?

☐ Very good; ☐ Good; ☐ Acceptable; ☐Poor; ☐Very poor

Q8. Do I have behavior problem?

☐ Yes; ☐ No

Q9. How is my self-esteem?

☐ Very good; ☐ Good; ☐ Acceptable; ☐Poor; ☐Very poor

Q10. How is my self-image?

☐ Very good; ☐ Good; ☐ Acceptable; ☐Poor; ☐Very poor

Q11. Do I have fear of being teased?

☐ Very often; ☐ Often; ☐ Occasionally; ☐Rarely; ☐ Never

Q12. Do I have symptoms of nasal regurgitation?

☐ Yes; ☐ No

Q13. Do I have symptoms of nasal obstruction?

☐ Yes; ☐ No

Q14. Do I suffer from rhinorrhea or allergic rhinitis?

☐ Yes; ☐ No

Q15. Do I have upper lip pain?

☐ Yes; ☐ No

Supplemental information: Questionnaire for Parents

Q1. Does your child's upper lip look symmetrical?

☐ Very Satisfied; ☐ Satisfied; ☐ Acceptable; ☐Unsatisfied; ☐Very unsatisfied

Q2. Does your child's upper lip border smooth (Without notching or bulging)?

☐ Very Satisfied; ☐ Satisfied; ☐ Acceptable; ☐Unsatisfied; ☐Very unsatisfied

Q3. How you feel the upper lip scar of your child?

☐ Very Satisfied; ☐ Satisfied; ☐ Acceptable; ☐Unsatisfied; ☐Very unsatisfied

Q4. How is the speech intelligibility of your child?

☐ Very Satisfied; ☐ Satisfied; ☐ Acceptable; ☐Unsatisfied; ☐Very unsatisfied

Q5. How is speech sound production of your child?

☐ Very Satisfied; ☐ Satisfied; ☐ Acceptable; ☐Unsatisfied; ☐Very unsatisfied

Q6. How is speech fluency of your child?

☐ Very Satisfied; ☐ Satisfied; ☐ Acceptable; ☐Unsatisfied; ☐Very unsatisfied

Q7. How is your child's social interaction?

☐ Very good; ☐ Good; ☐ Acceptable; ☐Poor; ☐Very poor

Q8. Does your child have behavior problem?

☐ Yes; ☐ No

Q9. How you describe the self-esteem of your child?

☐ Very good; ☐ Good; ☐ Acceptable; ☐Poor; ☐Very poor

Q10. How is self-image of your child?

☐ Very good; ☐ Good; ☐ Acceptable; ☐Poor; ☐Very poor

Q11. Does your child have fear of being teased?

☐ Very often; ☐ Often; ☐ Occasionally; ☐Rarely; ☐ Never

Q12. Does your child have symptoms of nasal regurgitation?

☐ Yes; ☐ No

Q13. Does your child have symptoms of nasal obstruction?

☐ Yes; ☐ No

Q14. Does your child suffered from rhinorrhea or allergic rhinitis?

☐ Yes; ☐ No

Q15. Does your child complained of upper lip pain?

☐ Yes; ☐ No
